# Flying Squirrel–associated Typhus, United States

**DOI:** 10.3201/eid0910.030278

**Published:** 2003-10

**Authors:** Mary G. Reynolds, John W. Krebs, James A. Comer, John W. Sumner, Thomas C. Rushton, Carlos E. Lopez, William L. Nicholson, Jane A. Rooney, Susan E. Lance-Parker, Jennifer H. McQuiston, Christopher D. Paddock, James E. Childs

**Affiliations:** * Centers for Disease Control and Prevention, Atlanta, Georgia, USA; † Marshall University, Huntington, West Virginia, USA; ‡ Atlanta I.D. Group, Atlanta, Georgia, USA; § West Virginia Division of Public Health, Charleston, West Virginia, USA; ¶ Georgia Division of Public Health, Atlanta, Georgia, USA

## Abstract

In March 2002, typhus fever was diagnosed in two patients residing in West Virginia and Georgia. Both patients were hospitalized with severe febrile illnesses, and both had been recently exposed to or had physical contact with flying squirrels or flying squirrel nests. Laboratory results indicated *Rickettsia prowazekii* infection.

Typhus fever from *Rickettsia prowazekii* infection is a severe and occasionally fatal disease in humans. Frequently referred to as epidemic typhus or louse-borne typhus, this disease can cause large epidemics when conditions are favorable for person-to-person spread of body lice (*Pediculus humanus humanus*). For the last few decades, reported outbreaks have been confined mainly to the cold mountainous regions of Africa and South America and have disproportionately affected impoverished and displaced communities (*1*).

Infections with *R. prowazekii* are rarely described in the United States. From 1976 to 2001, a total of 39 human *R. prowazekii* infections were documented in persons with no reported contact with body lice or persons with lice (*2*–*5*). Nearly all of these cases were in the eastern United States, and in approximately one third of cases, contact with flying squirrels (*Glaucomys* spp.) or with flying squirrel nests occurred before disease onset.

Flying squirrels are the only known vertebrate reservoir, other than humans, of *R. prowazekii*, and contact with these animals has been linked to most sporadic typhus cases in the United States. Interest in this disease was high in the 10 years after the first isolation of *R. prowazekii* from flying squirrels (*6*,*7*), but few cases have been reported since 1985. We describe two cases of flying squirrel–associated typhus that occurred in West Virginia and Georgia in 2002 and provide a contemporary summary of this disease in the United States.

## Case Reports

### West Virginia

During February 2002, a 44-year-old man in West Virginia arrived in the emergency department, with headache, fever, and chills. The patient also had hematuria, joint pain, discomfort on the left side of his abdomen, and vomiting. Laboratory findings included elevated levels of alanine transaminase (ALT) and aspartate transaminase (AST) (100 and 91 U/L, respectively), leukocyte count of 4.1 x 10^9^/L, platelet count of 249 x 10^9^/L, and erythrocyte sedimentation rate of 42 mm/h. The patient also had diverticulosis. A treatment regimen of levofloxacin and metronidazole was begun, and the patient was admitted to the hospital. The condition worsened, and an infectious disease specialist was consulted on day 4 of hospitalization. At this time, the patient was febrile (maximum temperature, 38°C), reported myalgia and malaise, and had mildly injected sclerae (without photophobia). AST and ALT levels remained slightly elevated. Levofloxacin and metronidazole were discontinued. Because the patient was a recreational hunter, serologic tests for Rocky Mountain spotted fever, ehrlichiosis, adenovirus, Lyme disease, and cytomegalovirus (CMV) were ordered, and doxycycline was given as empiric therapy. Serologic tests were negative for all agents. The patient was discharged on day 7 of hospitalization with a diagnosis of immunoglobulin (Ig) A nephropathy and hepatitis. At a follow-up visit (day 27 after illness onset), the patient still had myalgias, fatigue, and conjunctivitis, although his fever and abdominal pain had resolved. At this time, serologic testing for typhus group rickettsiae showed reactive IgM antibodies at a titer of 512 and IgG at a titer of <64. An additional serum sample obtained on day 53 after illness onset was tested at the Centers for Disease Control and Prevention (CDC) by indirect-immunofluorescence assay (IFA) and showed titers of IgM and IgG antibodies reactive with *R. prowazekii* of 128 and 1,024, respectively, indicating a recent acute infection.

During January, the patient had spent several nights in a hunting cabin in a rural area of Hardy County, West Virginia. Flying squirrels had infested the cabin every winter for several years; evidence of nesting materials and rodent feces in the attic and wall spaces was visible. The patient did not report seeing flying squirrels during visits to the cabin in 2002 but reported having removed rodent nesting materials and debris from a wall space 10–15 days before becoming ill.

### Georgia

During March 2002, a 57-year-old man from Fulton County, Georgia, received medical treatment for confusion associated with a febrile illness of approximately 1-week duration. The illness was characterized by rigors, malaise, myalgia, headache, vomiting, anorexia, and cyclic fever. During medical evaluation, dehydration, atrial fibrillation, and abnormal results for liver enzyme tests were also found. Pronounced neurologic symptoms with expressive aphasia, impaired coordination, and confusion were demonstrated. The cerebrospinal fluid (CSF) sample had normal protein and glucose levels, was negative for bacteria by routine culture, and was negative for herpes simplex virus by polymerase chain reaction (PCR). Cefepime, ampicillin, and gentamicin were given, and a presumptive diagnosis of bacterial meningitis was made. Additional history was obtained from the patient’s wife, who reported that 2 weeks before onset of symptoms, the patient had removed a flying squirrel carcass from the air intake chamber of the furnace in his office building’s basement. He had also taken the furnace filter outside and brushed it vigorously to remove dust and animal hair that had collected over the winter. The infectious disease specialist prescribed doxycycline for treatment of suspected flying squirrel–associated typhus after a history of contact with these animals was established. The patient reported no history of having had a similar unexplained illness in the past and reported no contact with human body lice or with persons with lice.

Serum specimens obtained on days 7, 13, and 23 after illness onset were evaluated at CDC for antibodies reactive with *R. prowazekii* antigens. The titer of specific IgG antibodies was 8,192 for all three specimens. The patient was discharged from the hospital on day 9 and demonstrated normal mental status by day 10 of discharge.

Serum samples were collected from the eight people who also worked in the patient’s office building. These specimens were screened by IFA for presence of antibody (IgG) reactive with typhus group rickettsiae antigens. All were negative (titer <16).

### Cases from 1985 to 2002

A review of records at CDC identified two additional cases of flying squirrel–associated typhus during 1985 to 2002 (Table). Typhus is not a nationally notifiable disease in the United States, and public health officials become aware of cases only when specialized confirmatory laboratory assays, performed at state health departments or CDC, are requested.

**Table:** Epidemiologic and clinical characteristics of flying squirrel–associated typhus fever in the United States, 1984–2002^a^

Characteristic	Case no.			
I	II^b^	III	IV
Sex	Male	Female	Male	Male
Age (y)	54	54	44	57
State of residence	Massachusetts	North Carolina	West Virginia	Georgia
Mo of onset	February 1998	September 1999	January 2002	March 2002
Flying squirrel contact	Y	Y	Y	Y
Serologic titer (no. d from onset)				
IgG *Rickettsia prowazekii*	4,096 (10)	nd	128 (53)	8,192 (7)
				8,192 (13)
	32,768 (38)			8,192 (23)
IgG *R. typhi*	nd	nd	<32 (53)	4,096 (7)
				4,096 (13)
				4,096 (23)
IgM *R. prowazekii*	nd	2,048 (18)	1,024 (53)	nd
IgM *R. typhi*	nd	512 (18)	1,024 (53)	nd
Symptoms				
Max fever (°C)	nr	40°C	38°C	40°C
Chills	Y	nr	Y	Y
Headache	Y	Y	Y	Y
Rash (type, location)	nr	Y (macular, trunk)	N	Y^c^

^a^Ig, immunoglobulin; Y, yes; N, no; nd, not done; nr, not recorded. ^b^Recurrent illness, first episode January 1999, flying squirrel contact, rash at that time. Other symptoms associated with both episodes. Serologic titer from second episode. ^c^Rash herpetic, not directly attributable to *R. prowazekii* infection but commonly seen in context of classic louse-borne epidemic typhus (*8*).

## Conclusions

Inhalation and transdermal or mucous membrane inoculation of infected louse feces are well-established routes of pathogen transmission during epidemics of human louse-borne typhus. The mechanism by which *R. prowazekii* is transmitted from flying squirrels to humans is less well understood. Various routes have been hypothesized, but none have been empirically established. Plausible mechanisms include inhalation or direct introduction (through mucous membrane or dermal abrasion) of infected feces from louse or flea ectoparasites of flying squirrels or through the bite of infected flea ectoparasites of flying squirrels (*9*). At least one species of flea ectoparasite (*Orchopeas howardii*) of flying squirrels is known to opportunistically bite humans and could serve as a bridge vector for transmission from flying squirrel to human. Rickettsiae transmission among captive flying squirrels, however, has only been demonstrated with a louse vector (*9*,*10*). While the exact mechanism of pathogen transmission has not yet been determined, the lack of detectable exposure to *R. prowazekii* in the household members or co-workers of documented cases (*3*,*5*) supports the idea that the risk for *R. prowazekii* infection after casual or indirect exposure to flying squirrels is low. Rather, existing evidence suggests that infection follows from close physical contact with flying squirrels or from exposure to a concentrated source of infectious materials (e.g., nests, dander, or infected ectoparasites).

Currently, no formal system for epidemic typhus surveillance exists in the United States, and diagnosis is hindered by the lack of rapid and reliable commercial tests. *R. prowazekii* infections can be confirmed by serologic testing, PCR, or organism culture. Commercial testing is, however, not widely available, and commercial serologic tests lack specificity because most detect antibodies reactive with a surrogate typhus-group rickettsial antigen (typically *R. typhi*). PCR (in conjunction with DNA sequencing) is a highly specific diagnostic tool (*11*) but has low sensitivity for commonly available clinical specimens, such as whole blood or serum. PCR may be used to greater effect on other clinical specimens (e.g., cerebrospinal fluid, lymphocytes, and skin biopsy) if they are collected and submitted for testing. Specific serologic and molecular testing is available at CDC for specimens submitted through state public health laboratories.

Sporadic epidemic typhus occurs in the United States, primarily during winter and spring, and in regions within the normal range of the southern flying squirrel (*Glaucomys volans*) (*12*). This illness can be severe, resulting in protracted hospital stays, particularly when diagnosis and appropriate treatment are delayed. In these two cases, treatment with tetracycline antibiotics was initiated after other broad-spectrum antibiotics were used. When therapy was changed to the appropriate antibiotic, the response was rapid. Tetracycline antibiotics are highly effective therapies for typhus (*13*). These cases underscore the importance of obtaining a thorough history of animal and arthropod contact in patients with acute febrile illness. Physicians and healthcare providers should remain alert to the signs and symptoms of epidemic typhus and be aware of appropriate diagnostic methods and antibiotic treatments (*13*,*14*).
